# Inquiry-based learning: A pedagogical tool to improving understanding of natural hazards

**DOI:** 10.4102/jamba.v14i1.1323

**Published:** 2022-12-06

**Authors:** Furqan I. Aksa

**Affiliations:** 1 Department of Geography Education, Faculty of Education, Universitas Samudra, Langsa, Indonesia

## Introduction

The frequency and impact of disaster events are increasing on a global scale (Coronese et al. 2018). Based on data released by the International Disaster Database (EM-DAT) in 2018, there were 315 disaster events worldwide, with the death toll reaching 11 804 people, and more than 68 million people were affected in various parts of the world (CRED 2018). Most of the fatalities were caused by earthquakes (45%) and floods (24%) (CRED 2018).

The fatalities are caused by a lack of knowledge and community preparedness in dealing with disasters (Escaleras, Anbarci & Register 2007). Education significantly improves preparedness (Battersby, Mitchell & Cutter 2011, Baytiyeh & Öcal 2016, Santos-Reyes et al. 2017). The Hyogo Framework for Action 2005–2015 (HFA), which the Sendai Framework has now replaced for Disaster Risk Reduction 2015–2030 (SFDRR), identified education as key to mitigating the impact of natural disasters (Kelman & Glantz 2015, UNISDR 2015). The HFA Priority 3 emphasises that disaster risk reduction (DRR) requires the use of knowledge, innovation and education to build a culture of safety and increase resilience (Kelman & Glantz 2015).

Geography education pays special attention to disaster studies because geography is an integrative discipline that brings together both the physical and human dimensions of the world in the study of people, places and environments (Mönter & Otto 2018, Sofiyan, Aksa & Saiman 2019). The Lucerne Declaration on Geographical Education for Sustainable Development of 2007 emphasises the importance of risk reduction and climate change being integrated into geography teaching around the world (Haubrich, Reinfried & Schleicher 2007). In Indonesia, disaster education has been integrated into the geography curriculum in universities since 2007 (Aksa 2019, Aksa et al. 2020a). These courses are taught following recommendations from the Geography College Leadership Forum and the Indonesian Geography Experts Association (IGI). The recommendation states that disaster education is essential to be taught to geography education students, aiming to prepare geography teachers who understand DRR.

However, even after 14 years, the programme has not been effective in increasing student capacity. This is evidenced by the findings of research conducted by Aksa et al. (2020a), which uses case studies at several universities in Indonesia which are located in disaster-risk areas. Using regression analysis, it was found that educational institutions were ineffective in increasing student capacity in DRR. This is presumably because the disaster education taught so far only forms conceptual knowledge (know-what) so that students do not know how to do something (know-how) (Aksa et al. 2020b, 2020a). This is because of the traditional learning model being used which emphasises conceptual knowledge. Aksa et al. (2020b) stated that disaster learning taught in geography study programmes only forms conceptual understanding. Also, students are only taught about the conceptual framework of risk reduction and hazards (Aksa et al. 2020b). Selby and Kagawa (2012) pointed out that learning on DRR carried out in Indonesia still focuses on memorisation and facts, and not skills or attitudes. For example, in implementing the DRR curriculum in Indonesia, learning is carried out only by remembering facts (Selby & Kagawa 2012). Teachers are not trained in developing creative and innovative learning methodologies (Selby & Kagawa 2012, Aksa et al. 2020b). Disaster preparedness simulations are carried out very infrequently (Aksa et al. 2020b).

Conceptual knowledge is ineffective in increasing motivation to take preparedness actions (Shaw et al. 2004, Muzenda-Mudavanhu, Manyena & Collins 2016). Furthermore, it is not relevant to the objectives of disaster education as described in the SFDRR, which emphasises knowledge, skills and attitudes (Kelman & Glantz 2015). Parham et al. (2021) reported that the benefits of disaster education conducted in various countries, including Indonesia, were not felt outside the classroom and did not significantly increase students’ awareness of disaster risk. This is because the pedagogical approach currently used does not provide opportunities for students to be involved in participatory decision-making exercises (Aksa et al. 2020b, Kamil et al. 2020a, 2020b, Parham et al. 2021).

In addition, disaster learning in the geography study programme is not following the guidelines for disaster education in universities developed by the Directorate General of Higher Education (2019) Indonesia, which emphasises conceptual learning (knowing), practice (doing) and application in the community. Disaster education in higher education curriculum aims to produce resilient graduates to disasters (Kementerian Riset Teknologi dan Pendidikan Tinggi 2019).

It is imperative to redesign the education model in the geography study programme. Therefore, this research aims to introduce the inquiry-based learning (IBL) model to increase students’ in-depth understanding of hazards. The novelty is to introduce a collaborative and participatory learning model. It begins by providing a philosophical foundation for IBL and provides an example of a project on studying flood hazards.

The example of the geo-inquiry project is expected to be used in disaster learning in the Indonesian geography education study programme. The application of geo-inquiry as a pedagogical tool is essential because geography students are prospective teachers who play an important role in providing an understanding of natural hazards. Teachers have been recognised to play an essential role in transferring knowledge to students and their parents (Sakurai et al. 2018; Shiwaku & Fernandez 2011). They also contribute significantly to increasing the resilience of communities (Oktari et al. 2015). In addition, teachers, schools and other stakeholders play prominent roles in building a disaster-resilient community.

The role of geography teachers as a catalyst in disseminating knowledge to the public was proven during the 2004 Indian Ocean tsunami disaster. A 10-year-old student from England who was on vacation with his family on Phuket Beach, Thailand, managed to save hundreds of lives of people who were vacationing on the beach. The student from England could identify signs of a tsunami by looking at the seawater suddenly receding and foam bubbles appearing in the middle of the ocean. This knowledge was obtained from geography lessons at his school 2 weeks before the tsunami disaster occurred (Gregg et al. 2006).

The United Nations Office for Disaster Risk Reduction (UNISDR) (2015) stated that educating prospective teachers is the most effective, inexpensive, long-term and sustainable approach. This is believed to improve their skills and capacity when teaching risk reduction and increase students’ resilience (Chen, Yu & Chen et al. 2012; Johnson & Ronan 2014).

## Inquiry-based learning in disaster

The author chose IBL because the United Nations Educational, Scientific and Cultural Organization (UNESCO) recommends this approach in disaster education. In addition, inquiry has the advantages of a project-based learning (PBL) model; IBL encourages students to seek information independently through investigation to become more independent, critical and analytical.

**FIGURE 1:**
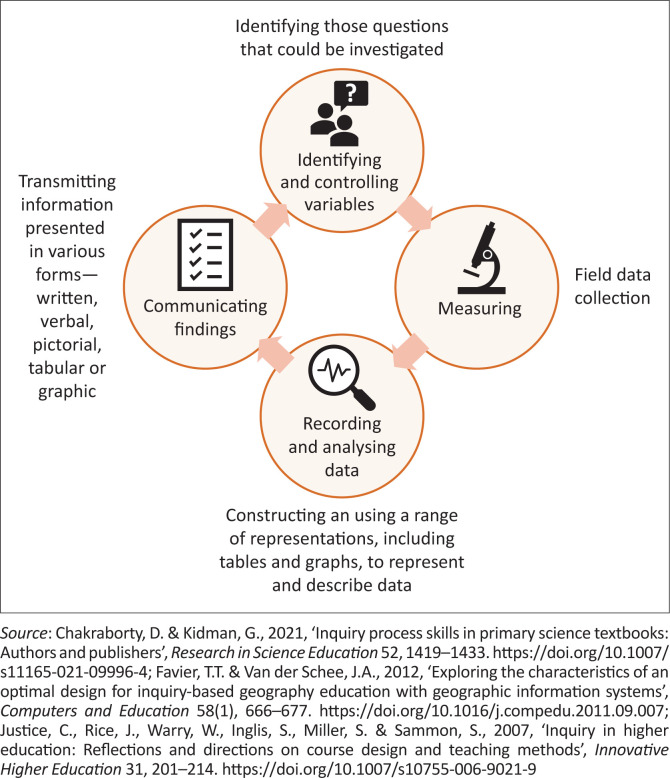
Inquiry-based learning.

Inquiry-based learning is a student-centred approach (Gholam 2019; Kidman & Casinader 2017; Oberle 2020). The idea of involving students in inquiry originated from Dewey (1938), who believed that the learning environment should enable them to solve problems collaboratively (Dewey 1938). Inquiry-based learning is an increasingly used approach in geography educational curricula worldwide (Justice et al. 2021; McNeal et al. 2020; Oberle 2020; Seow, Chang & Neil Irvine 2019). The approach consists of several stages, namely identifying and controlling variables; measuring, recording and analysing data; as well as communicating findings (Figure 1).

Furthermore, the approach promotes students to ask questions, identify problems, plan and conduct research, predict results and provide conclusions (Gouramanis & MoralesRamirez 2021). This pedagogical approach can improve higher-order cognition (Aubrey & Riley 2019). It is also relevant to several theories, such as the ‘invitational theory’, which seeks to empower and build confidence in learners to investigate and explain phenomena sources (Favier & Van Der Schee 2012; Haigh 2011; Karvánková et al. 2017; Seow et al. 2019; Yani 2021). Several studies have found that inquiry learning can increase awareness of global climate change (GCC), which in turn can increase awareness and action to reduce GCC (Namdar 2018).

Inquiry-based learning is the best practice in geography education (Bednarz 2016). Inquiry-based learning in disaster learning can improve students’ cognition through continuous reflection (Cooper et al. 2020; Justice et al. 2021). In Singapore, inquiry is considered a ‘signature pedagogy’ that can socialise students into the practices, concepts and values of the geography discipline (Seow et al. 2019). Inquiry-based learning emphasises a participatory approach, an essential element in DRR education (Hosseini & Izadkhah 2020; Kamil et al. 2020a; Yani 2021). The participatory approach in DRR education is very important to be believed to be effective in increasing community capacity (Gaillard & Mercer 2013). Several studies have proven that a participatory approach can significantly increase community understanding regarding disaster management (Oktari 2019).

Inquiry-based learning allows students to ask questions, negotiate, explore questions and share other scenarios to develop their critical understanding of phenomena on the earth’s surface (Chakraborty & Kidman 2021; Favier & Van Der Schee 2012; Justice et al. 2007). Bednarz (2016) highlighted that IBL is essential in geography learning. It will increase the capacity of learners in self-management as well as build their investigative skills (Bednarz 2016).

This learning is believed to increase student capacity in effectively conducting investigations. Selby and Kagawa (2012) outlined several effective and efficient learning methods in disaster education, namely brainstorming, role-playing, experimental learning and inquiry. The inquiry learning that emphasises case research is believed to increase the in-depth understanding of disasters (Gouramanis & MoralesRamirez 2021). The core of PBL involves students constructing knowledge by asking them to complete projects and develop products (Guo et al. 2020; Karvánková et al. 2017; Roberts et al. 2010). The main difference between PBL and IBL is that IBL is a learning approach that directs students to find knowledge, ideas and information through investigation.

Project-based learning has several advantages: focusing on learning objectives, participation, collaboration and product creation (Guo et al. 2020). However, PBL has several weaknesses also, including the need for preparation and a long time to process. The success of implementing PBL in disaster studies is strongly influenced by students’ activeness in exploring the material.

Inquiry-based learning emphasises critical and analytical thinking processes to find the answer to a question (Justice et al. 2007, 2021, Kidman & Casinader 2017). Learning using the inquiry model is believed to be more effective in increasing capacity. Namdar (2018) reported that the application of learning using the inquiry model effectively increases teacher understanding in Turkey about GCC. They also stated they were better prepared to teach GCC in their classrooms (Namdar 2018).

In addition, the inquiry learning model includes the model recommended by UNESCO in disaster education (Selby & Kagawa 2012). According to UNESCO, inquiry builds students’ understanding of the causes, nature and impact of hazards while also developing various competencies and skills to enable them to contribute proactively to disaster prevention and mitigation (Selby & Kagawa 2012). Inquiry is an approach that makes students actively participate in learning, so it has the potential to be a catalyst, UNESCO also states (Selby & Kagawa 2012).

It was stated that inquiry could increase the understanding of risk reduction (Cooper et al. 2020; Gouramanis & MoralesRamirez 2021; Yani 2021). The model is believed to be very appropriate and effective in universities. Inquiry-based learning is related to constructivist theory, which suggests that knowledge is acquired through active involvement. Also, inquiry is considered an attempt to translate constructivist theory into practice.

This learning model emphasises the active involvement of students in conducting hazard risk assessments. The direct experiential learning increases students’ understanding of hazards (Zavar & Nelan 2020). However, there is currently limited literature on the application of IBL in disaster studies, specifically in Indonesia. The following section will introduce a proposal that can be applied to geography students.

## Exemplary geo-inquiry projects: Flood risk module in Indonesia

The following section will explain an example of applying geo-inquiry to the topic of flooding in Langsa City, Indonesia. The geo-inquiry process is a learning approach that aims to develop the skills, knowledge and attitudes of a geographer. The geo-inquiry process provides a systematic way to investigate and understand the world through patterns, processes and interactions between humans and nature (Oberle 2020; Oberle et al. 2019).

The difference between the developed model with the previous model lies in the geographical perspective in the form of a spatial aspect as the basis for conducting investigations. Another difference between the old approach and this new approach is that students formulate their questions, not being chosen by lecturers or outside experts. Lecturers only act as facilitators in the learning.

**FIGURE 2:**
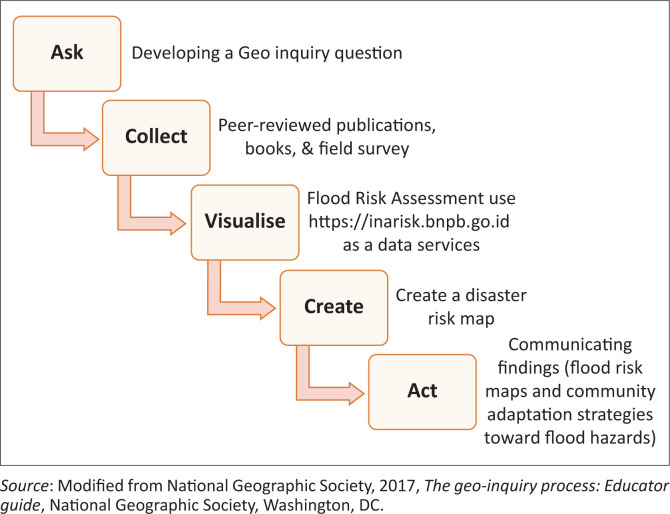
The geo-inquiry process.

The geo-inquiry process consists of five steps, namely: ask, collect, visualise, create and act (Figure 2). The geo-inquiry project begins with the ‘Ask’ phase, where the lecturer provides initial information to make it easier for students to formulate questions. The lecturer conveys data and facts regarding the danger of flooding in Langsa City, Indonesia. Geo-inquiry begins by formulating questions related to flooding problems in Langsa City. The lecturer explained that Langsa City is one of the areas in Indonesia that has a flood risk index. The results of the disaster risk analysis conducted by the National Disaster Management Agency (BNPB) in 2015 showed that Langsa City had a high category flood risk index (National Disaster Management Agency 2015). Students are interested in the problem of routine flooding that always occurs in Langsa City every year. The geo-inquiry question formulated is ‘what can they do to reduce the risk of flooding in Langsa City?’

To answer these questions, students proceed to the ‘Collect’ phase. At this stage, students will learn how to collect data to answer flood problems in Langsa City, Indonesia. Data were collected through a systematic literature review, research, surveys, observations, interviews and photos and videos. Students can also design data collection tools according to the problem to be solved.

After data collection, students ‘Visualise’ by exploring spatial patterns of flood hazards, compiling infographics and determining the correct type of map and visualisation technique to describe flood risk in Langsa City. During visualisation, students can use the https://inarisk.bnpb.go.id portal as a data source. InaRisk is a portal created by Indonesia’s BNPB. This portal presents disaster risk assessment results using ArcGIS servers as data services. The data presented contains the coverage of the hazard area, affected population, potential physical and economic loss, as well as environmental damage. InaRisk plays an important role in providing spatial data to students regarding hazards in an area.

In the ‘Create’ stage, students compile a flood risk map, flood risk infographic data and a map of flood evacuation routes. In the ‘Act’ phase, students present the results of flood risk analysis, evacuation route maps and community adaptation strategies to flood hazards using communication tools such as videos and posters to stakeholders.

The effectiveness of geo-inquiry learning is measured by authentic assessment. The difference between the old approach and geo-inquiry assessment is that this approach uses authentic assessment, which changes the culture of objective tests that only focus on knowledge into a more comprehensive assessment based on student performance results. The authentic assessment aims to integrate what students learn in the classroom with the problems faced by professionals in the real world (Fook 2010; Villarroel et al. 2018).

Authentic assessment is carried out using a rubric that involves lecturers and stakeholders. A rubric is commonly used in authentic assessment (Ghosh et al. 2016; Nkhoma et al. 2020). Rubrics as authentic assessment instruments are ideal for improving higher-order cognitive skills (Fook 2010; Ghosh et al. 2016; Nkhoma et al. 2020). In addition, other empirical results show that rubrics play an essential role in authentic assessment regardless of level or discipline (Ghosh et al. 2016; Nkhoma et al. 2020).

The rubric used to measure the effectiveness of geo-inquiry learning consists of the following criteria:

1. Students succeeded in explaining specifically the risk of flood disaster in Langsa City (completed with a risk map).
2. The novelty of community adaptation ideas to flood hazards in Langsa City.
3. The ability to clearly articulate the results of the geo-inquiry project using posters or videos.

In detail, an example of a rubric for assessing student learning outcomes in the flood module is given in Table 1.

**TABLE 1:** Rubric for assessing student learning outcomes in the flood module.

Score	Description
**Criteria 1:** Students succeeded in explaining specifically the risk of flood disaster in Langsa City (completed with a risk map)	
3	Explains an accurate and complete description (complete with a flood risk map in Langsa City)
2	Explains a partial but accurate description of the risk of flooding in Langsa City.
1	Provide incomplete, unclear and inaccurate descriptions of flood risk in Langsa City.
**Criteria 2:** The novelty of community adaptation ideas to flood hazards in Langsa City, Indonesia	
3	Provide innovative, up-to-date ideas that the community can apply to reduce the risk of flood disasters (complete with a map of flood evacuation routes)
2	Provide ideas on adaptation strategies that are less innovative but can be applied by the community to reduce flood risk
1	Provide ideas for adaptation strategies that are not innovative and cannot be applied by the community to reduce flood risk.
**Criteria 3:** The ability to clearly articulate the results of the geo-inquiry project	
3	Students can clearly articulate the results of the geo-inquiry project using clear and informative language, equipped with posters and videos.
2	Students can clearly articulate the results of the geo-inquiry project using clear language but are not equipped with posters and videos.
1	Students are not able to articulate clearly the results of the geo-inquiry project and are not equipped with posters and videos.

Authentic assessment has been shown to positively impact student learning (Fook 2010; Villarroel et al. 2018). Authentic assessment can improve students’ learning motivation, self-regulation, higher-order cognitive skills and metacognition (Ashford-Rowe, Herrington & Brown 2014; Fook 2010; Villarroel et al. 2018).

## Conclusion

The effectiveness of disaster education is strongly influenced by the learning model used. The disaster education taught in the geography education programme still focuses on memorising concepts and facts. This article introduces the geo-inquiry pedagogical approach in disaster education. The geo-inquiry consists of several stages: ask, collect, visualise, create and act. In the ‘ask’ phase, students develop a geo-inquiry question. In the ‘collect’ stage, students collect data to answer research questions. In the ‘visualise’ phase, students visualise by doing a flood risk assessment using https://inarisk.bnpb.go.id as a data service. Lastly, students create a disaster risk map and ‘act’, namely communicating findings (flood risk map) and community adaptation strategies toward flood hazards. The geo-inquiry learning model is expected to increase students’ understanding of flood hazards and actions that can be taken to reduce flood risk.
